# Non-dipping pulse rate and chronic changes of the kidney in patients with chronic kidney disease

**DOI:** 10.3389/fcvm.2023.911773

**Published:** 2023-02-20

**Authors:** Rina Oba, Go Kanzaki, Kotaro Haruhara, Takaya Sasaki, Yusuke Okabayashi, Kentaro Koike, Nobuo Tsuboi, Takashi Yokoo

**Affiliations:** Division of Nephrology and Hypertension, Department of Internal Medicine, The Jikei University School of Medicine, Tokyo, Japan

**Keywords:** autonomic imbalance, ambulatory blood pressure monitoring, cardiorenal syndrome, chronic kidney disease, microanatomical structure, non-dipping pulse rate

## Abstract

**Introduction:**

An insufficient decrease in nocturnal pulse rate (PR), non-dipping PR, reflects autonomic imbalance and is associated with cardiovascular events and all-cause mortality. We aimed to investigate the clinical and microanatomical structural findings associated with the non-dipping PR status in patients with chronic kidney disease (CKD).

**Methods:**

This cross-sectional study included 135 patients who underwent ambulatory blood pressure monitoring and kidney biopsy concurrently at our institution between 2016 and 2019. Non-dipping PR status was defined as (daytime PR-nighttime PR)/daytime PR <0.1. We compared clinical parameters and microstructural changes in the kidney between patients with and without non-dipping PR, including 24 h proteinuria, glomerular volume, and Mayo Clinic/Renal Pathology Society Chronicity Score.

**Results:**

The median age was 51 years (interquartile range: 35–63), 54% of which were male, and the median estimated glomerular filtration rate was 53.0 (30.0–75.0) mL/min/1.73 m^2^. Non-dipping PR status was observed in 39 patients. Patients with non-dipping PR were older and had worse kidney function, higher blood pressure, greater prevalence of dyslipidemia, lower hemoglobin levels, and a larger amount of urinary protein excretion than patients with dipping PR. Patients with non-dipping PR had more severe glomerulosclerosis, interstitial fibrosis, tubular atrophy, and arteriosclerosis. In the multivariable analysis, the severe chronic changes of the kidney were associated with non-dipping PR status after adjusting for age, sex, and other clinical parameters (odds ratio = 20.8; 95% confidence interval, 2.82–153; *P* = 0.003).

**Conclusion:**

This study is the first to indicate that non-dipping PR is significantly associated with chronic microanatomical changes in the kidneys of patients with CKD.

## 1. Introduction

Blood pressure (BP) and pulse rate (PR) physiologically decrease by 10–20% at night due to the relative dominance of the parasympathetic nervous system (1, 2). Non-dipping, the phenomenon of insufficient nocturnal BP and PR decline (defined as less than 10% from day to night), reflects autonomic imbalance.

Abnormal circadian rhythms of BP, including non-dipping and nocturnal rising BP, are established risk factors for hypertensive target organ damage and cardiovascular events (3–5). In this regard, ambulatory blood pressure monitoring (ABPM) is useful for predicting long-term kidney and cardiovascular outcomes in patients with chronic kidney disease (CKD) (6, 7). We reported cross-sectional studies using ABPM and kidney biopsy specimens, which showed that chronic kidney biopsy findings were significantly associated with both daytime and nighttime hypertension in patients with CKD (8) and patients with IgA nephropathy (9). In addition, several experimental models have demonstrated that tubulointerstitial damage induces salt-sensitive hypertension (10, 11). These studies suggest the possibility that intrarenal mechanisms regulate systemic BP.

Likewise, recent studies have reported that non-dipping PR is associated with cardiovascular events and all-cause mortality in the general population and in patients with hypertension, acute myocardial infarction, and type 2 diabetes (2, 12–16). These studies have shown that non-dipping PR status is associated with several clinical factors, including age, sex, body mass index (BMI), hypertension (HTN), and diabetes mellitus (DM) (12, 14). However, little is known about non-dipping PR in patients with CKD. A recent cross-sectional study showed that the prevalence of non-dipping PR was higher in CKD and hemodialysis patients than in the general population (14, 17). Previous clinical and experimental studies have suggested that autonomic cardiovascular alterations occur in the presence of CKD, contributing to cardiovascular morbidity and mortality (18, 19). Although ABPM recordings provide PR together with BP measurements, studies regarding ambulatory PR data are limited, and the association between non-dipping PR and microanatomical structural changes in kidney biopsies is unclear.

In the present study, we investigated the clinical and microanatomical characteristics of non-dipping PR in Japanese patients with CKD using both ABPM and kidney biopsy specimens and aimed to identify pathophysiological factors associated with non-dipping PR status.

## 2. Materials and methods

### 2.1. Subjects

**FIGURE 1:**
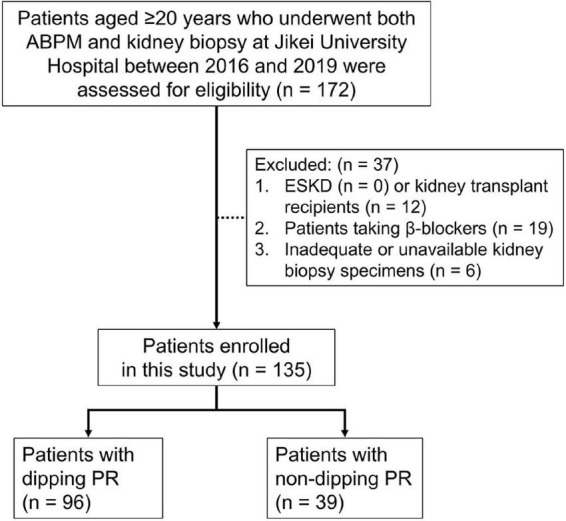
Flow diagram of study participants. One hundred and seventy-two patients aged ≥20 years who underwent ABPM and kidney biopsy at our institution between March 2016 and March 2019 were assessed for eligibility. Of these patients, 37 were excluded from this study due to the exclusion criteria. ABPM, ambulatory blood pressure monitoring; ESKD, end-stage kidney disease; PR, pulse rate.

Patients who underwent both ABPM and kidney biopsy during the same admission period from March 2016 to March 2019 at Jikei University Hospital, Tokyo, Japan, were included in the present cross-sectional study. A kidney biopsy was performed at the time of diagnosis and, in case of repetition, only the first diagnostic biopsy was included. All subjects were diagnosed with primary or secondary glomerular disease by kidney biopsy. We excluded patients who fall within the following criteria: aged <20 years, end-stage kidney disease, kidney transplant recipients, patients taking β-blockers, those with less than 5 glomeruli in the kidney biopsy (8, 20), and those with no histopathological data available (Figure 1).

This study was approved by the Ethics Review Board of The Jikei University School of Medicine (33–265, 10,883) and was carried out according to the Declaration of Helsinki. Because this was a cross-sectional study, information on the research plan was proposed and an opportunity to opt out was provided; therefore, individual informed consent was waived for this study. All patients provided their written informed consent for kidney biopsy.

### 2.2. Clinical measurements

General demographic data, including age, sex, height, body weight, type of antihypertensive medication, frequency of DM and frequency of dyslipidemia, were obtained from medical records at the time of diagnostic kidney biopsy. Laboratory measurements included hemoglobin, creatinine, estimated glomerular filtration rate (eGFR), uric acid, low-density lipoprotein cholesterol (LDL-C), high-density lipoprotein cholesterol (HDL-C), triglyceride (TG), urinary β2-microglobulin, and 24 h proteinuria. Urine was collected for 24 h, and dietary sodium intake was assessed using 24 h urinary sodium excretion. Estimated protein intake was estimated from 24 h urinary urea excretion according to Maroni’s formula (21), normalized to ideal body weight, and expressed as g/kg/day. Ideal body weight was defined as a BMI of 22 kg/m^2^, as previously reported (22).

Diabetes mellitus was defined as a HbA1c value > 6.5% (National Glycohemoglobin Standardization Program) or the use of antidiabetic medications. Dyslipidemia was defined as LDL-C ≥ 140 mg/dL, HDL-C < 40 mg/dL, TG ≥ 150 mg/dL, or the use of antihyperlipidemic agents (23). BMI was defined as body weight divided by the square of height, and eGFR was calculated using the following formula for Japanese subjects: eGFR (mL/min/1.73 m^2^) = 194 × Cr^–1.094^ × age^–0.287^ (× 0.739 for women) (24).

In addition, patients were classified into four CKD prognostic groups (low risk, moderately increased risk, high risk and very high risk) by glomerular filtration rate (GFR) and proteinuria categories according to the KDIGO (Kidney Disease Improving Global Outcomes) 2012 Clinical Practice Guidelines for the Evaluation and Management of Chronic Kidney Disease (Supplementary Figure 1) (25).

### 2.3. ABPM measurements and definition of non-dipping status

Ambulatory blood pressure monitoring analyses were performed using a TM-243 device (A&D, Tokyo, Japan). Measurements were taken at 30-min intervals from 06:00 to 22:00, whereas measurements were taken at 1-h intervals from 22:00 to 06:00. The daytime and nighttime were defined as the awake and sleeping periods, respectively, which were determined by the activity records of the patients (26). According to the Japanese Society of Hypertension Guidelines for the Management of Hypertension (JSH 2019) (27), 24 h hypertension was defined as an average 24 h systolic BP (sBP) of ≥130 mmHg and/or an average 24 h diastolic BP (dBP) of ≥80 mmHg. Daytime hypertension was defined as an average diurnal sBP ≥135 mmHg and/or an average diurnal dBP ≥85 mmHg. Nighttime hypertension was defined as an average nocturnal sBP ≥120 mmHg and/or an average nocturnal dBP ≥70 mmHg. Mean arterial pressure (MAP) was defined as dBP plus pulse pressure divided by three. The PR and BP drop rates were defined as (daytime PR-nighttime PR)/daytime PR and (daytime sBP-nighttime sBP)/daytime sBP, respectively. Non-dipping PR status was defined as a PR drop rate <0.1, whereas non-dipping BP status was defined as a BP drop rate <0.1 (12–15, 17, 28).

### 2.4. Microanatomical structural findings

Kidney biopsy specimens were obtained using percutaneous needle biopsy, after which tissues were embedded in paraffin, cut into 3-μm sections, and stained with hematoxylin and eosin, periodic acid–Schiff, Masson’s trichrome, and periodic acid silver–methenamine. These specimens were evaluated by light microscopy, immunostaining, and electron microscopy in routine diagnostic pathological assessments. The number and percentage of glomeruli with segmental and/or global sclerosis were also recorded. In addition, the mean glomerular volume (GV) was estimated from the measured mean glomerular tuft area as follows: GV = (glomerular tuft area)^3/2^ × β/d, where β is a dimensionless shape coefficient (β = 1.382 for spheres), and d is the size distribution coefficient used to adjust for variations in glomerular size (*d* = 1.01) (29). The severity of arteriosclerosis was graded based on the most severe lesions by comparing the thickness of the intima with that of the media in the same segment of the vessel; it was graded on a scale of 0 to 2 (grade 0: absence of intimal thickening, 1: intimal thickening <thickness of media, 2: intimal thickening ≥ thickness of media) (8, 30). The percentage of segmental and/or global glomerulosclerosis (GS%) and interstitial fibrosis and/or tubular atrophy (IF/TA) was semiquantitatively scored on a scale of 0 to 3 (score 0 for <10%, 1 for 10% to 25%, 2 for 26% to 50%, and 3 for >50%). From these scores, the total renal chronicity score was determined according to the Mayo Clinic/Renal Pathology Society Chronicity Score (31) to grade the overall severity of chronic lesions as minimal (0–1 total score), mild (2–4 total score), moderate (5–7 total score), and severe (≥8 total score). Arteriosclerosis was scored from 0 to 1 (0: intimal thickening <thickness of media; 1: intimal thickening ≥ media thickness) in this scoring system. Interstitial fibrosis (IF) and tubular atrophy (TA) usually occur together, but rarely develop independently of each other (31). IF and TA scores are considered equal to the IF/TA score in this study.

### 2.5. Statistical analyses

Continuous variables are presented as medians and interquartile ranges (IQRs) or numbers with percentages in parentheses. Differences in continuous variables were analyzed using the Mann–Whitney U test or Kruskal–Wallis test, as appropriate, and those in categorical variables were analyzed using the chi-square test or Fisher’s exact test according to the sample size. The Dunn–Bonferroni test was used as a *post hoc* analysis for the Kruskal–Wallis test. Spearman’s correlation analysis was used to analyze the correlations among the variables. Multivariable logistic regression analysis was performed to identify independent determinants of non-dipping PR status in patients with CKD. All covariates except sex, the presence of DM, the renal chronicity score grades and the use of antihypertensive medications were treated as continuous variables in the multivariable analyses. Missing values are excluded and addressed for each variable. Statistical significance was defined as a two-sided *P* < 0.05. All statistical analyses were performed with SPSS v.25.0 (IBM Corp., Armonk, NY, USA).

## 3. Results

### 3.1. Subject selection

A total of 172 patients aged ≥20 years underwent both ABPM and kidney biopsy at our institution between March 2016 and March 2019. Of these patients, 37 were excluded from this study due to the following exclusion criteria: kidney transplant recipients, taking β-blockers, inadequate glomerular number or unavailable kidney biopsy specimens. After exclusion, 135 patients were enrolled, of which 39 (28.9%) patients were in the non-dipping PR group and 96 (71.1%) patients were in the dipping PR group (Figure 1).

### 3.2. Baseline demographic and clinical characteristics

Tables 1, 2 summarize the demographic, clinical and laboratory data of the patients. The median age was 51 years (IQR: 35–63), 54% of which were male, and the median eGFR at diagnosis was 53.0 (30.0–75.0) mL/min/1.73 m^2^. The proportions of each GFR category were as follows: G1 7.4%, G2 32.6%, G3a 16.3%, G3b 20.0%, G4 13.3%, and G5 10.4%.

**TABLE 1:** Clinical and demographic characteristics of the subjects according to the dipping and non-dipping pulse rate pattern.

	All (*n* = 135)	Dipping (*n* = 96)	Non-dipping (*n* = 39)	*P*-value
Age (years)	51 (35–63)	46 (33–59)	62 (49–74)	**<0.001**
Male, *n* (%)	73 (54)	50 (52)	23 (59)	0.47
eGFR (mL/min/1.73 m^2^)	53.0 (30.0–75.0)	59.0 (36.0–79.0)	36.0 (14.0–53.0)	**<0.001**
Diabetes, *n* (%)	23 (17)	14 (15)	9 (23)	0.23
Dyslipidemia, *n* (%)	90 (67)	58 (60)	32 (82)	**0.02**
BMI (kg/m^2^)	22.2 (20.4–24.7)	22.0 (20.2–24.6)	22.9 (20.8–25.3)	0.43
Hb (g/dL)	13.0 (11.1–14.7)	13.6 (11.8–14.9)	11.2 (10.1–13.1)	**<0.001**
Anemia, *n* (%)	56 (41)	29 (30)	27 (69)	**< 0.001**
UA (mg/dL)	6.3 (5.1–7.3)	6.1 (5.0–7.1)	7.0 (5.2–7.5)	0.05
HbA1c (%)^a^	5.6 (5.4–6.0)	5.6 (5.4–6.0)	5.7 (5.4–6.1)	0.90
LDL-C (mg/dL)^b^	121 (101–142)	121 (101–137)	120 (94–165)	0.53
HDL-C (mg/dL)^c^	60 (50–73)	60 (49–75)	62 (51–70)	0.63
TG (mg/dL)^d^	134 (102–183)	136 (104–196)	117 (95–164)	0.10
EPI/IBW (g/kg/day)	0.8 (0.7–1.0)	0.8 (0.7–1.0)	0.8 (0.6–1.0)	0.13
NaCl (g/day)	6.1 (4.4–8.5)	5.8 (4.4–7.8)	7.0 (4.3–9.8)	0.18
UPE (g/day)	0.9 (0.4–2.1)	0.7 (0.3–1.6)	1.8 (0.6–4.3)	**0.002**
u-β2MG (μg/L)^e^	128 (55–2,465)	84 (0–452)	1,363 (119–12,095)	**<0.001**
Prognosis of CKD by GFR and proteinuria categories				**<0.001**
Low risk, *n* (%)	9 (6)	9 (9)	0 (0)	–
Moderately increased risk, *n* (%)	15 (11)	12 (13)	3 (8)	–
High risk, *n* (%)	40 (30)	33 (34)	7 (18)	–
Very high risk, *n* (%)	71 (53)	42 (44)	29 (74)	–

eGFR, estimated glomerular filtration rate; BMI, body mass index; Hb, hemoglobin; UA, uric acid; LDL-C, low-density lipoprotein cholesterol; HDL-C, high-density lipoprotein cholesterol; TG, triglyceride; EPI/IBW, estimated protein intake divided by ideal body weight; NaCl, sodium intake; UPE, urinary protein excretion; u-β2MG, urinary β2-microglobulin; CKD, chronic kidney disease; GFR, glomerular filtration rate. Values are presented as medians and interquartile ranges (IQRs) or numbers with percentages in parentheses. The bold values indicate statistically significant.

^a^11 cases are missing.

^b^2 cases are missing.

^c^4 cases are missing.

^d^2 cases are missing.

^e^22 cases are missing.

**TABLE 2:** Ambulatory blood pressure monitoring measurements of the subjects according to the dipping and non-dipping pulse rate pattern.

	All (*n* = 135)	Dipping (*n* = 96)	Non-dipping (*n* = 39)	*P-*value
Antihypertensive agent	72 (53)	47 (49)	25 (64)	0.11
Ca channel blocker, *n* (%)	41 (30)	21 (22)	20 (51)	**0.003**
ACE-I/ARB, *n* (%)	55 (41)	35 (36)	20 (51)	0.11
Diuretics, *n* (%)	15 (11)	7 (7.3)	8 (21)	**0.03**
Alfa blocker, *n* (%)	1 (0.7)	0 (0.0)	1 (2.6)	0.29
24 h sBP (mmHg)	125 (114–141)	122 (113–135)	141 (125–152)	**<0.001**
Diurnal sBP (mmHg)	128 (116–143)	124 (115–138)	142 (125–155)	**<0.001**
Nocturnal sBP (mmHg)	118 (108–133)	113 (107–128)	133 (114–146)	**<0.001**
24 h dBP (mmHg)	76 (70–84)	75 (69–83)	81 (72–92)	**0.03**
Diurnal dBP (mmHg)	77 (71–87)	76 (71–84)	80 (73–93)	0.05
Nocturnal dBP (mmHg)	70 (64–78)	68 (62–75)	78 (66–88)	**0.001**
24 h MAP (mmHg)	93 (85–102)	90 (84–100)	102 (90–112)	**0.001**
Diurnal MAP (mmHg)	94 (86–105)	91 (86–102)	99 (92–115)	**0.003**
Nocturnal MAP (mmHg)	86 (78–96)	84 (77–91)	99 (81–109)	**<0.001**
24 h PR (bpm)	72 (66–78)	72 (66–78)	72 (67–78)	0.95
Diurnal PR (bpm)	74 (68–79)	74 (68–80)	72 (68–79)	0.34
Nocturnal PR (bpm)	63 (57–69)	60 (55–66)	68 (63–74)	**<0.001**
PR drop (%)	14.3 (8.6–19.4)	17.0 (13.0–21.0)	6.8 (5.8–8.1)	**<0.001**
BP drop (%)	7.9 (1.8–12.1)	8.3 (2.5–12.4)	5.7 (0.0–11.7)	0.12
24 h HTN, *n* (%)	66 (49)	38 (40)	28 (72)	**<0.001**
Diurnal HTN, *n* (%)	59 (44)	31 (32)	28 (72)	**<0.001**
Nocturnal HTN, *n* (%)	80 (59)	51 (53)	29 (74)	**0.02**
BP non-dipping, *n* (%)	83 (61)	56 (58)	27 (69)	0.24

Ca, calcium; ACE-I, angiotensin-converting enzyme inhibitor; ARB, angiotensin II receptor blocker; sBP, systolic blood pressure; dBP, diastolic blood pressure; MAP, mean arterial pressure; PR, pulse rate; HTN, hypertension; BP, blood pressure. Values are presented as medians and interquartile ranges (IQRs) or numbers with percentages in parentheses. BP drop rate was defined as (daytime sBP-nighttime sBP)/daytime sBP. The bold values indicate statistically significant.

Patients with non-dipping PR were significantly older and had worse kidney function at baseline. Dyslipidemia was more prevalent among patients with non-dipping PR than among those with dipping PR. Patients with non-dipping PR had significantly lower levels of hemoglobin, a greater amount of urinary protein excretion, and a greater amount of urinary β2-microglobulin than patients with dipping PR. Twenty-nine out of 39 (74%) patients with non-dipping PR were classified as “very high risk” in the CKD prognostic categories, whereas 42 out of 96 (44%) patients with dipping PR were classified in the same category. Of note, other characteristics, including sex, frequencies of DM, BMI, serum uric acid, cholesterol levels, estimated protein intake, and sodium intake were not statistically different between the two groups (Table 1). Patients with non-dipping PR had higher 24 h/diurnal/nocturnal BP parameters and a higher prevalence of 24 h/diurnal/nocturnal HTN. 24 h PR and diurnal PR were not statistically different between the groups. In addition, the presence of non-dipping BP was not associated with non-dipping PR (*P* = 0.24, Table 2).

### 3.3. Association between non-dipping PR and kidney function

**FIGURE 2:**
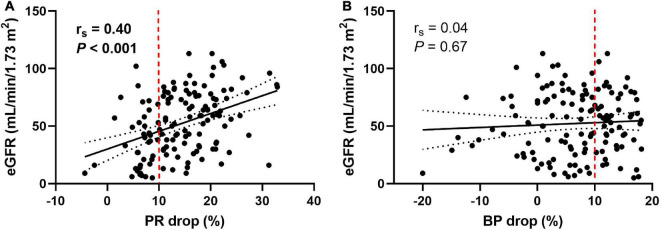
Pulse rate and blood pressure drop in relation to the baseline eGFR. The pulse rate drop rate showed a positive linear correlation with baseline eGFR **(A)**, whereas the blood pressure drop rate did not show a significant correlation with baseline eGFR **(B)**. Measures of association were tested by Spearman’s correlation analysis. Solid lines indicate lines of best fit. Dotted lines show 95% confidence intervals. The dashed red line represents 10% of the pulse rate drop **(A)** and the blood pressure drop **(B)**. eGFR, estimated glomerular filtration rate; PR, pulse rate; BP, blood pressure.

As shown in Table 1, patients with non-dipping PR had worse kidney function than those with dipping PR; however, there were no differences in baseline eGFR between patients with and without non-dipping BP. When we analyzed the association between PR or BP drop rate and kidney function as continuous variables, the PR drop rate exhibited a positive linear correlation with baseline eGFR (r_*s*_ = 0.40, *P* < 0.001, Figure 2A), whereas the BP drop rate was not significantly correlated with baseline eGFR (r_*s*_ = 0.04, *P* = 0.67, Figure 2B).

### 3.4. Comparison of microanatomical characteristics

Table 3 lists the characteristics of microanatomical structural changes in the kidney. Kidney biopsies contained a median of 29 (21–39) glomeruli, and the median GS% and IF/TA were 20.0% (5.9–46.2) and 10.0% (5.0–30.0), respectively. Patients with non-dipping PR had significantly higher GS%, IF/TA, and more severe arteriosclerosis (*P* < 0.001, *P* < 0.001, and *P* = 0.02, respectively) than patients with dipping PR; therefore, patients with non-dipping PR had a higher renal chronicity score than those without it (*P* < 0.001). GV was not significantly different between the groups. The proportion of the histopathological diagnosis was as follows: IgA nephropathy, 54 patients (40%); nephrosclerosis, 13 patients (9.6%); focal segmental glomerulosclerosis, 13 patients (9.6%); membranous nephropathy, 10 patients (7.4%); minimal change disease, 8 patients (5.9%); diabetic nephropathy, 7 patients (5.2%); tubulointerstitial nephritis, 6 patients (4.5%); membranoproliferative glomerulonephritis, 3 patients (2.2%); and others, 21 patients (15.6%) (Supplementary Table 1).

**TABLE 3:** Microanatomical structural characteristics of the subjects according to the dipping and non-dipping pulse rate pattern.

	All (*n* = 135)	Dipping (*n* = 96)	Non-dipping (*n* = 39)	*P-*value
GV (×10^6^μm^3^)	2.3 (1.8–3.4)	2.3 (1.8–3.3)	2.6 (1.7–3.8)	0.43
GS (%)	20.0 (5.9–46.2)	13.1 (5.0–31.0)	46.2 (13.9–64.5)	**<0.001**
IF/TA (%)	10.0 (5.0–30.0)	10.0 (5.0–20.0)	30.0 (10.0–60.0)	**<0.001**
Arteriosclerosis grade				**0.02**
0: No intimal thickening	29 (21)	23 (24)	6 (15)	–
1: Intima < Media	34 (25)	29 (30)	5 (13)	–
2: Intima > Media	72 (53)	44 (46)	28 (72)	–
Renal chronicity score				**<0.001**
Minimal (score 0–1)	40 (30)	36 (38)	4 (10)	–
Mild (score 2–4)	36 (26)	29 (30)	7 (18)	–
Moderate (score 5–7)	39 (29)	25 (26)	14 (36)	–
Severe (score ≥ 8)	20 (15)	6 (6)	14 (36)	–

GV, mean glomerular volume; GS, segmental and/or global glomerulosclerosis; IF/TA, interstitial fibrosis and/or tubular atrophy. Values are presented as medians and interquartile ranges (IQRs) or numbers with percentages in parentheses. The bold values indicate statistically significant.

### 3.5. Comparison of four categories regarding PR and BP dipping status

When we classified subjects as having or not having BP and/or PR non-dipping into four categories (Table 4), the proportion of very high risk CKD prognostic categories increased in the following order: BP/PR dipping, BP non-dipping/PR dipping, BP dipping/PR non-dipping, and BP/PR non-dipping. The BP/PR non-dipping group had significantly lower eGFR than the BP/PR dipping and BP non-dipping/PR dipping groups (adjusted *P* = 0.004 and *P* = 0.002, respectively). The GS% was significantly higher in the BP/PR non-dipping group than in the BP/PR dipping and BP non-dipping/PR dipping groups (adjusted *P* = 0.006 and *P* < 0.001, respectively). The IF/TA ratio was significantly higher in the BP/PR non-dipping group than in the BP/PR dipping group (adjusted *P* = 0.02).

**TABLE 4:** Clinical and microanatomical characteristics of the subjects according to the dipping and non-dipping blood pressure/pulse rate pattern.

	DBP/DPR (*n* = 40)	NBP/DPR (*n* = 56)	DBP/NPR (*n* = 12)	NBP/NPR (*n* = 27)	*P-*value
**Clinical parameters**					
Age (years)	44 (33–55)	51 (33–60)	64 (49–73)	60 (49–76)	**0.001^abcd^**
Male, *n* (%)	25 (63)	25 (45)	4 (33)	19 (70)	**0.04**
eGFR (mL/min/1.73 m^2^)	59.0 (39.0–78.8)	61.0 (35.0–80.5)	42.5 (16.3–56.0)	33.0 (13.0–53.0)	**<0.001^abd^**
Prognosis of CKD by GFR and proteinuria categories					**0.01^abd^**
Low risk, *n* (%)	3 (7)	6 (11)	0 (0)	0 (0)	–
Moderately increased risk, *n* (%)	8 (20)	4 (7)	2 (17)	1 (4)	–
High risk, *n* (%)	12 (30)	21 (37)	2 (17)	5 (18)	–
Very high risk, *n* (%)	17 (43)	25 (45)	8 (66)	21 (78)	–
**Microanatomical structural parameters**					
GV (×10^6^μm^3^)	2.4 (1.9–3.4)	2.2 (1.6–3.1)	2.0 (1.6–3.4)	2.7 (1.9–3.9)	0.34
GS (%)	15.5 (5.2–36.9)	13.1 (3.8–30.1)	39.4 (11.8–53.6)	54.5 (20.0–73.0)	**<0.001^abd^**
IF/TA (%)	10.0 (5.0–20.0)	10.0 (5.0–25.0)	30.0 (11.3–47.5)	30.0 (5.0–60.0)	**0.004^ab^**
Arteriosclerosis grade					0.11
0: No intimal thickening	10 (25)	13 (23)	1 (8)	5 (19)	–
1: Intima < Media	13 (32)	16 (29)	2 (17)	3 (11)	–
2: Intima > Media	17 (43)	27 (48)	9 (75)	19 (70)	–
Renal chronicity score					**<0.001^abd^**
Minimal (score 0–1)	14 (35)	22 (39)	0 (0)	4 (15)	–
Mild (score 2–4)	15 (38)	14 (25)	3 (25)	4 (15)	–
Moderate (score 5–7)	7 (17)	18 (32)	8 (67)	6 (22)	–
Severe (score ≥ 8)	4 (10)	2 (4)	1 (8)	13 (48)	–

DBP, dipping blood pressure; DPR, dipping pulse rate; NBP, non-dipping blood pressure; NPR, non-dipping pulse rate; eGFR, estimated glomerular filtration rate; CKD, chronic kidney disease; GV, mean glomerular volume; GS, segmental and/or global glomerulosclerosis; IF/TA, interstitial fibrosis and/or tubular atrophy. Values are presented as medians and interquartile ranges (IQRs) or numbers with percentages in parentheses. BP drop rate was defined as (daytime systolic BP-nighttime systolic BP)/daytime systolic BP. The bold values indicate statistically significant.

^a^Kruskal–Wallis test with Dunn–Bonferroni test.

^b^Adjusted *P* < 0.05, DBP/DPR vs. NBP/NPR.

^c^Adjusted *P* < 0.05, DBP/DPR vs. DBP/NPR.

^d^Adjusted *P* < 0.05, NBP/DPR vs. NBP/NPR.

### 3.6. Clinical and microanatomical factors associated with non-dipping PR status

Univariable and multivariable adjusted logistic regression analyses were performed to identify the independent determinants of non-dipping PR status in this population (Table 5). As per univariable analysis, non-dipping PR status was associated with age, baseline eGFR, urinary protein excretion, MAP, and moderate and severe renal chronicity score groups. As per multivariable analysis, the severe chronic changes of the kidney were significantly associated with non-dipping PR status after adjustment for age, sex, and previously reported covariates, including eGFR, 24 h proteinuria, the presence of DM, BMI, and MAP (odds ratio = 20.8; 95% confidence interval, 2.82–153; *P* = 0.003). Moreover, we added the use of antihypertensive agents to the age/sex-adjusted multivariable model to adjust for each effect of antihypertensive agents, which did not change the association between the renal chronicity score and non-dipping PR status (Supplementary Table 2).

**TABLE 5:** Univariable and multivariable logistic regression analysis for determinants of non-dipping pulse rate.

	Univariable		Multivariable	
	OR (95% CI)	*P*-value	Adjusted OR (95% CI)	*P*-value
Age (years)	1.05 (1.03–1.08)	**<0.001**	**1.05 (1.02–1.08)**	**0.003**
Sex (Male)	1.32 (0.62–2.81)	0.47	0.90 (0.33–2.46)	0.84
eGFR (mL/min/1.73 m^2^)	0.97 (0.95–0.98)	**<0.001**	1.00 (0.98–1.03)	0.76
UPE (g/day)	1.15 (1.01–1.31)	**0.03**	1.09 (0.92–1.30)	0.31
Diabetes	1.76 (0.69–4.48)	0.24	0.61 (0.18–2.14)	0.44
BMI (kg/m^2^)	1.02 (0.94–1.11)	0.59	0.96 (0.86–1.06)	0.40
24 h MAP (mmHg)	1.05 (1.02–1.08)	**0.001**	1.04 (0.99–1.09)	0.06
Renal chronicity score				
Minimal (score 0–1)	Reference	-	Reference	-
Mild (score 2–4)	2.17 (0.58–8.15)	0.25	1.63 (0.35–7.56)	0.54
Moderate (score 5–7)	5.04 (1.48–17.1)	**0.01**	3.06 (0.55–17.0)	0.20
Severe (score ≥8)	21.0 (5.14–85.8)	**<0.001**	**20.8 (2.82–153)**	**0.003**

OR, odds ratio; CI, confidence interval; eGFR, estimated glomerular filtration rate; UPE, urinary protein excretion; BMI, body mass index; MAP, mean arterial pressure. All covariates except sex, the renal chronicity score and the presence of diabetes were treated as continuous variables. The bold values indicate statistically significant.

## 4. Discussion

Two major findings novel to the present study are as follows. First, the non-dipping PR status was observed in approximately 30% of patients with CKD, and the highest proportion of patients with CKD with non-dipping PR was in the “very high risk” category by the KDIGO GFR and proteinuria categories. Second, even after adjustment for clinical parameters, severe chronic changes in kidney microanatomical structure were significantly associated with insufficient decline in nocturnal PR, which is reported to indicate autonomic nervous system dysfunction. To our knowledge, this is the first study to investigate the association between non-dipping PR and clinical and microanatomical factors in patients with CKD. We identified microanatomical structural changes in the kidney responsible for non-dipping PR; therefore, it may contribute to understanding the pathophysiology behind the autonomic imbalance in patients with CKD.

Although the pathophysiology of the development / progression of CKD and autonomic disorders has not been fully elucidated, a CKD animal model showed that central and peripheral circadian clocks were disrupted in mice with CKD, and the study suggested that circadian disruption accelerated CKD progression, resulting in a vicious cycle (32). In addition, cardiovascular autonomic dysfunction is an important complication of CKD (18, 33). There is a bidirectional interaction between the heart and kidneys, and the dysfunction of one organ may induce the dysfunction of the other, which is called cardiorenal syndrome. Cardiorenal syndrome is frequently complicated by anemia, which is also called cardiorenal anemia syndrome (34). Anemia was more prevalent among patients with non-dipping PR than among those with dipping PR in this study. Several mechanisms, including autonomic, neural, hormonal, and vascular mechanisms, have been implicated in its pathogenesis (35). A high nocturnal heart rate increases the mechanical load and shear stress on the endothelium, leading to endothelial dysfunction and predisposition to arteriosclerosis (17, 36). In accordance with this vascular mechanism, we observed more severe chronic microvascular changes, including arteriosclerosis, IF/TA, and glomerulosclerosis, in kidney biopsies of patients with non-dipping PR than in those with dipping PR.

Moreover, we hypothesize that intrarenal renin-angiotensin system (RAS) activation may be one of the underlying mechanisms of the association between non-dipping PR and chronic changes (GS% and IF/TA) in this study. Urinary angiotensinogen is an established surrogate marker for intrarenal RAS activity, and a previous pathological study showed that urinary angiotensinogen levels were correlated with the severity of GS% and IF/TA (37). Another study reported that urinary angiotensinogen levels had a significant positive association with an increase in nighttime heart rate in patients with CKD (38). Intrarenal RAS activation increases intraglomerular pressure and induces proteinuria, followed by progressive kidney damage and activation of the sympathetic nervous system (38). We observed an association between non-dipping PR and chronic changes irrespective of patient age, kidney function, and use of antihypertensives. In the participants of this study with circadian disruption, intrarenal RAS may be activated, leading to the further formation of chronic microvascular changes.

The renal chronicity score, which is derived from the sum of semiquantitative assessments of chronic changes, has been proposed as a uniform approach to grade chronic changes in kidney biopsy specimens (31). Chronic changes are generally irreversible and are important for predicting the prognosis and response to treatment. A prospective cohort study including native kidney biopsies with various kidney diseases showed that each component of the renal chronicity score was associated with the progression of kidney disease, even after adjusting for clinical predictors, including eGFR and proteinuria. In addition, this study validated that the renal chronicity score was independently associated with kidney failure (39). In this study, we observed a significant association between renal chronicity score and non-dipping PR after adjustments for clinical covariates. Patients with non-dipping PR had higher renal chronicity scores, and the largest proportion belonged to the very high risk group of the KDIGO categories in this study. We speculate that patients with non-dipping PR may exhibit poorer kidney outcomes than those without dipping PR, although this cannot be proven from our study. Future longitudinal studies are needed.

Notably, there was no association between non-dipping PR and non-dipping BP, which is consistent with previous studies (12, 14, 15). A randomized double-blind study investigating the effects of melatonin suggested that BP and PR are regulated by different mechanisms (40). In addition, when we divided subjects into the abovementioned four categories according to BP/PR non-dipping status, the proportion of very high risk CKD prognostic categories tended to increase in the following order: BP/PR dipping, BP non-dipping/PR dipping, BP dipping/PR non-dipping, and BP/PR non-dipping (Table 4). The risk of all-cause mortality is reported to increase in the following order: sole non-dipping BP, sole non-dipping PR, and a combination of non-dipping BP and non-dipping PR when a combination of dipping BP and dipping PR is a reference group (12). Another study showed that the combination of BP and PR non-dipping resulted in a significant synergistic increase in the risk of cardiovascular events (13). However, in this study we did not investigate cardiovascular or all-cause mortality.

This study had several limitations. First, the study design was cross-sectional, and the causal relationship between non-dipping PR and microanatomical parameters could not be proven. In this regard, we assessed non-dipping PR status in relation to CKD prognostic categories by KDIGO and renal chronicity score, as these parameters are established prognostic factors. Second, ABPM was performed once during the hospitalization period, and these results might be different from ABPM performed in outpatient clinics; however, restricted diet and physical activity during hospitalization can minimize the effect of interpersonal differences, which could be an advantage at the same time. Third, we did not consider the physical activity of the patients, smoking habits, food intake, and sleep quality. Finally, the present study was limited to a single center with Japanese patients with CKD who underwent kidney biopsies, making it difficult to generalize these results to all patients with CKD.

Despite these limitations, this study is the first to investigate the association between a flattened PR circadian rhythm and microanatomical structural findings of the kidney. This study supported the hypothesis that the bidirectional interactions between chronic changes in the kidney and circadian disruption fall into a vicious cycle in patients with CKD. We speculate that patients with CKD and non-dipping PR have an additional risk of cardiovascular events and poor kidney outcomes compared to patients with CKD and dipping PR. Non-dipping PR is an important but neglected parameter, and only limited studies focusing on this topic have emerged in recent decades (41). A new attempt to monitor non-dipping PR with wearable devices has been proposed in a recent review (41). Therefore, we await future studies with these new devices to further investigate the effects of non-dipping PR on CKD.

## 5. Conclusion

Non-dipping PR status was observed in 28.9% of patients with CKD, and the majority of patients with non-dipping PR belonged to the “very high risk” category by the KDIGO GFR and proteinuria categories. In conclusion, this study indicates that a flattened PR circadian rhythm is associated with chronic changes in kidney microanatomical structure.

## Data availability statement

The raw data supporting the conclusions of this article will be made available by the authors, without undue reservation.

## Ethics statement

The studies involving human participants were reviewed and approved by the Ethics Review Board of The Jikei University School of Medicine (33–265, 10,883). Written informed consent for participation was not required for this study in accordance with the national legislation and the institutional requirements.

## Author contributions

RO and GK: conceptualization, formal analysis, investigation, data curation, and writing—original draft preparation. RO, KH, and GK: methodology. TY: resources and supervision. RO, GK, KH, TS, YO, KK, NT, and TY: writing—review and editing. RO: visualization. GK: project administration. All authors contributed to data interpretation and approved the final version of the manuscript.
