# Supplementary material for: Non-dipping pulse rate and chronic changes of the kidney in patients with chronic kidney disease
Source: Front Cardiovasc Med. 2023 Feb 20;10:911773. doi: 10.3389/fcvm.2023.911773 (PMC9986326; doi:10.3389/fcvm.2023.911773)
Supplement: Supplementary file 1 [file Data_Sheet_1.docx]

Supplementary Material

# Supplementary Figure

**Supplementary Figure 1. Prognosis of Chronic Kidney Disease by glomerular filtration rate (GFR) and Proteinuria Categories: KDIGO (Kidney Disease Improving Global Outcomes) 2012.**

**
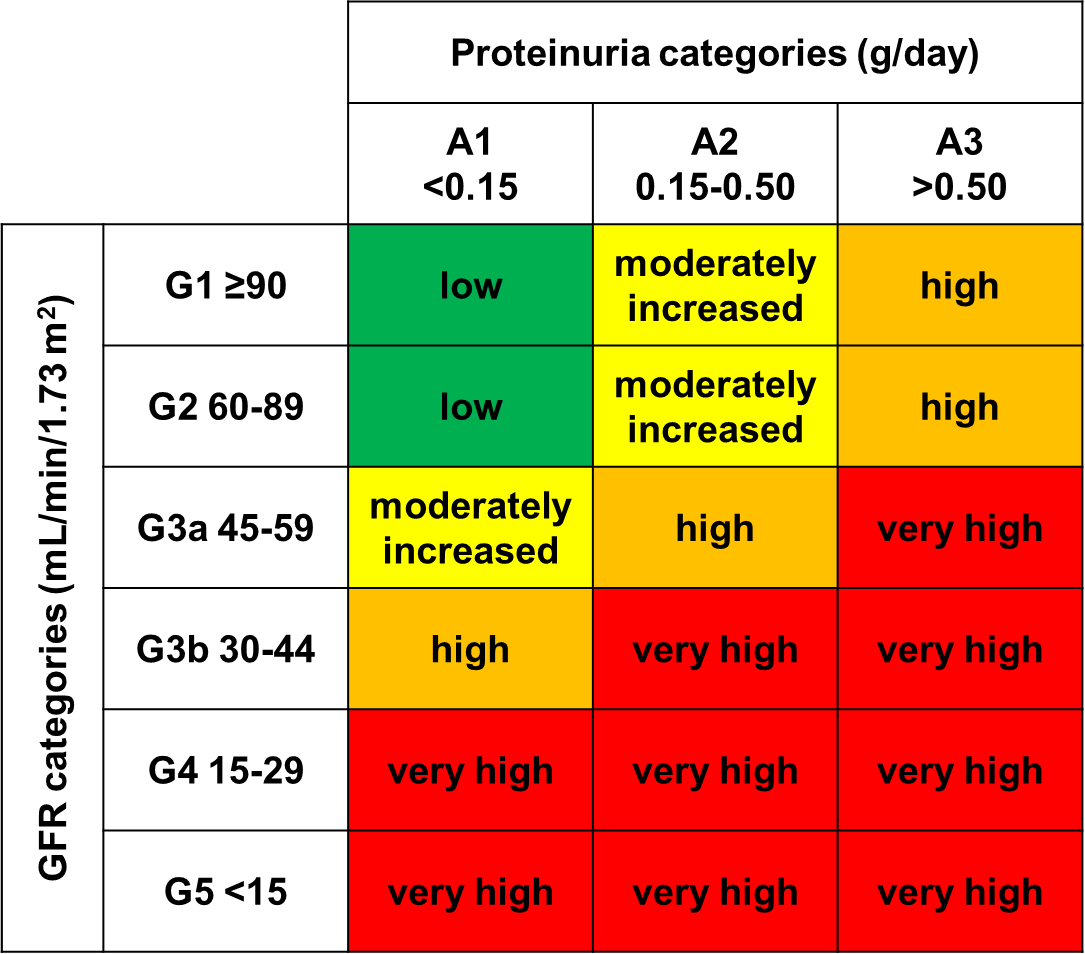
**

Green: low risk; Yellow: moderately increased risk; Orange: high risk; Red: very high risk.

*GFR, glomerular filtration rate.*

# Supplementary Tables

**Supplementary Table 1. Pathological diagnosis of the subjects according to the dipping and non-dipping pulse rate pattern.**

| **Pathological diagnosis,**  **n (%)** | **All**  (n = 135) | **Dipping**  (n = 96) | **Non-dipping**  (n = 39) |
| --- | --- | --- | --- |
| **IgAN** | 54 (40) | 44 (46) | 10 (25.6) |
| **Nephrosclerosis** | 13 (9.6) | 6 (6.2) | 7 (17.9) |
| **FSGS** | 13 (9.6) | 9 (9.4) | 4 (10.3) |
| **MN** | 10 (7.4) | 8 (8.3) | 2 (5.1) |
| **MCD** | 8 (5.9) | 5 (5.2) | 3 (7.7) |
| **DMN** | 7 (5.2) | 1 (1.0) | 6 (15.4) |
| **TIN** | 6 (4.5) | 5 (5.2) | 1 (2.6) |
| **MPGN** | 3 (2.2) | 1 (1.0) | 2 (5.1) |
| **Others** | 21 (15.6) | 17 (17.7) | 4 (10.3) |

*IgAN, IgA nephropathy; FSGS, focal segmental glomerulosclerosis; MN, membranous nephropathy; MCD, minimal change disease; DMN, diabetic nephropathy; TIN, tubulointerstitial nephritis; MPGN, membranoproliferative glomerulonephritis.*

**Supplementary Table 2. Multivariable logistic regression analysis for determinants of non-dipping pulse rate.**

|  | **Multivariable** | |
| --- | --- | --- |
|  | **Adjusted OR (95% CI)** | ***P* value** |
| **Age (years)** | **1.05 (1.02–1.08)** | **0.003** |
| Sex (Male) | 1.01 (0.40–2.56) | 0.99 |
| **Renal Chronicity Score** |  | - |
| Minimal (score 0–1) | reference | **-** |
| Mild (score 2–4) | 1.79 (0.40–7.96) | 0.45 |
| Moderate (score 5–7) | 2.74 (0.64–11.7) | 0.17 |
| **Severe (score ≥8)** | **18.5 (3.54–96.7)** | **0.001** |
| Ca channel blocker | 1.53 (0.52–4.52) | 0.44 |
| ACE-I/ARB | 0.83 (0.30–2.29) | 0.71 |
| Diuretics | 2.53 (0.57–11.2) | 0.22 |

Age was treated as continuous variables. *Ca, calcium; ACE-I, angiotensin-converting enzyme inhibitor; ARB, angiotensin Ⅱ receptor blocker; OR, odds ratio; CI, confidence interval.*
